# Exploring learning engagement among Spanish undergraduates in Asia: the chain mediating roles of flow experience and self-determined motivation in short video platform use

**DOI:** 10.3389/fpsyg.2026.1709572

**Published:** 2026-02-09

**Authors:** Lin Liang, Shasha Wang

**Affiliations:** 1 Henan University of Science and Technology, Luoyang, China; 2 Universitat Autònoma de Barcelona, Barcelona, Spain

**Keywords:** flow experience, language learning, learning engagement, self-determined motivation, short video platforms

## Abstract

**Introduction:**

This paper examines the effects of short video platform use on learning engagement among undergraduate students majoring in Spanish in Asia, focusing on the chained mediating roles of flow experience and self-determined motivation. With the rapid growth of short video platforms, particularly in Asia, such platforms have become an integral part of university students’ daily lives. Although short videos are easily accessible due to low usage barriers, they may also cause problems such as distraction and time wastage, making it necessary to investigate their role in educational contexts.

**Methods:**

To explore the impact of both functional and recreational uses of short video platforms on learning engagement and the underlying psychological mechanisms, a questionnaire-based survey was conducted among 412 undergraduate Spanish majors from China, Japan, South Korea, and Vietnam.

**Results:**

The findings indicate that functional use of short video platforms plays a significant role in enhancing learning engagement, with a total effect of 0.677. This effect is achieved through the reinforcement of flow experience (0.815, p < 0.001) and self-determined motivation (0.326, p < 0.001). Moreover, flow experience and self-determined motivation form a chained mediating mechanism, in which the mediating effect of flow experience on motivation related to recreational use accounts for 26.6% of the total effect (0.180).

**Discussion:**

These results provide theoretical insights for educators on how short video platforms can be effectively used to facilitate student learning engagement. At the same time, they highlight the potential negative impact of excessive recreational use of such platforms in learning design. Future research should further examine the effects of different types of short video content and extend the investigation to other language-learning environments and cultural contexts, thereby enriching the applicability of short videos in educational research.

## Introduction

In the last 10 years, the short-form video platforms (including Tik Tok/Douyin, Kuaishou, and Instagram Reels) have been well integrated into the lives of college students, especially in Asia. With few barriers of entry due to personalized suggestions, bite-sized content and high levels of interactivity, these sites offer further opportunities of acquisition of knowledge and at the same time present certain challenges like abusive time and the restructuring of cognitive attention (Chen et al., 2023; Levey, 2025). As proposed in the research related to education, it has been demonstrated that brief and short video contents can positively influence the learning process and that they can contribute to a better usability of obtaining the information when it comes to course design, such as evidence shows that a short video helps boost the viewing time and academic performance in an online flipped classroom (Rith et al., 2025; Sisi and Zainudin, 2025). Nonetheless, according to other researchers, the overconsumption or addiction to the short videos can affect the deep learning and academic dedication (Ye et al., 2022; Ye et al., 2024). Short videos may also be used as an addition to other materials in language teaching settings, which facilitate listening, speaking and cultural exposure. Their fragmented and entertainment-focused nature however can also have detrimental effect on those foreign language learning activities that involve sustained attention and metacognition regulation.

It is against this background that the study being presented focuses on the undergraduate Spanish majors in Asia. This population is at an extreme phase of the second language acquisition that requires a significant amount of input and drilling, and, at the same time, is both a regular use and an activist producer of short video content. Respectively, it is of significant theoretical and practical interest to explore how the use of short video platform can affect their learning experience and the underlying psychological processes, i.e., flow experience and self-determined motivation. Theoretically, this work combines the media psychology (short video use) with motivational and experiential frames (flow theory by Csikszentmihalyi, 1988; self-determination theory by Deci and Ryan, 2012), which will fill the empirical void on the processes by which short videos can influence the outcome of language learning. Practically, the results can serve as advice to the university-level instructors of foreign languages, curriculum developers, and school administrators in the way they may utilize short videos to assist their teaching process and reduce the negative implications of entertainment-driven usage (Wang et al., 2024).

It is necessary to investigate the connection between the use of short videos and learning activities by differentiating between various categories and degrees of the usage. Available empirical evidence has shown that instructors can use short videos as a part of instruction design (teacher-created or course-provided explanatory videos) that can considerably contribute to the active participation of students in viewing, classroom interaction, and the partial acquisition of knowledge (Priyakanth et al., 2021; Shen, 2024). This is an indication that the application of short videos in the instructional setting, where they are used with specific learning objectives can be an important means of promoting learning interaction. In comparison, short videos which are not instructional and those with entertainment purposes, often represented by fragmented consumption (navigating feeds) are often linked to distraction, less time studying, and negative associations with learning outcomes (Asif and Kazi, 2024; Liu et al., 2022). Thus, it is necessary to differentiate the functional and entertainment-rise-based usage of short videos because both types can have the opposite influence on the course of learning.

According to Uses and Gratifications Theory (Katz et al., 1973) and available existing empirical evidence, various kinds of the use of short video platforms can have different effects on the engagement in the learning process. The functional use is usually characterized by clear learning objectives and the systematic content in the educational context. As an example, Priyakanth et al. (2021) discovered that instructional short videos provided by teachers had a significant positive effect on the participation of students. On the other hand, entertainment usage may have no steep learning objectives. Asif and Kazi (2024) established that this use is more likely to distract one and decrease the learning time. Thus, our hypothesis is as follows: *H1:* The functional use of short video platforms is positively related to the learning engagement of undergraduate Spanish students and the recreational use of video platforms is negatively related to the learning engagement of undergraduate Spanish students.

Moreover, the flow viewpoint shows that short video sites have the potential to cause high levels of attentional capture and distorted perceptions of time in its users because of its high-immediacy feedback and immersive characteristics. These experiences typically have positive links with stickiness and repeated use of the platform and long distance of use in entertainment consumption (Levey, 2025; Yan et al., 2023). This state may encourage solid engagement with learning exercises in the context of education where content short videos clearly meet both conditions of challenge and skill matching coupled with clear goals, which lead to the flow of activities. On the other hand, when flow is mainly present during the process of non-academic scrolling, it diverts conscious attention and lessens the learning process (Özhan and Kocadere, 2020; Tomas and Poroto, 2023). Another theory of flow introduced by Csikszentmihalyi (1990) depicts that a person can go into a high level of focus when the challenge of the activity is corresponding to his or her level of skill. Wang and Huang (2022) discovered that an environment with well-designed content can result in the creation of learning flow in the short video learning. In the meantime, the study by Özhan and Kocadere (2020) affirmed that a learning experience flow improves learning interactions in educational settings. According to these theoretical reasons and research conclusions: *H2:* The flow-experience intermediates the connection between the use of a short video platform and learning involvement.

Alternatively, according to the self-determination theory, both situational supports, such as relevance, autonomy, and competence, and intrinsic motivation, such as interest and interest-driven proactive learning, determine the engagement in learning (Deci and Ryan, 2012). In the cases where short video platforms are learning tools that promote the competence of learners (via instant positive feedback and rehearsal), the autonomy (via on demand content selection and selection), and relatedness (via peer sharing and recognition), the latter stimulate intrinsic and self-determined motivation, which, in turn, reinforces the level of engagement in learning. On the other hand, fragmented and externally motivated platform use can support self-determination and deter long-term engagement of learning (Huang et al., 2019; Shang et al., 2025). The Self-Determination Theory (Deci and Ryan, 2000) identifies three fundamental psychological factors necessary to motivate the intrinsic motivation, namely competence, autonomy, and connection. Huang et al. (2019) discovered that through the support of such needs provided by short video platforms, students can be motivated to learn. Junco (2012) also affirmed that the mechanism of motivation of self-determination is also vital in maintaining long-term learning participation. Thus: *H3:* Self-determined motivation mediates between the use of short video platforms and engaging in learning activities.

**Figure 1:**
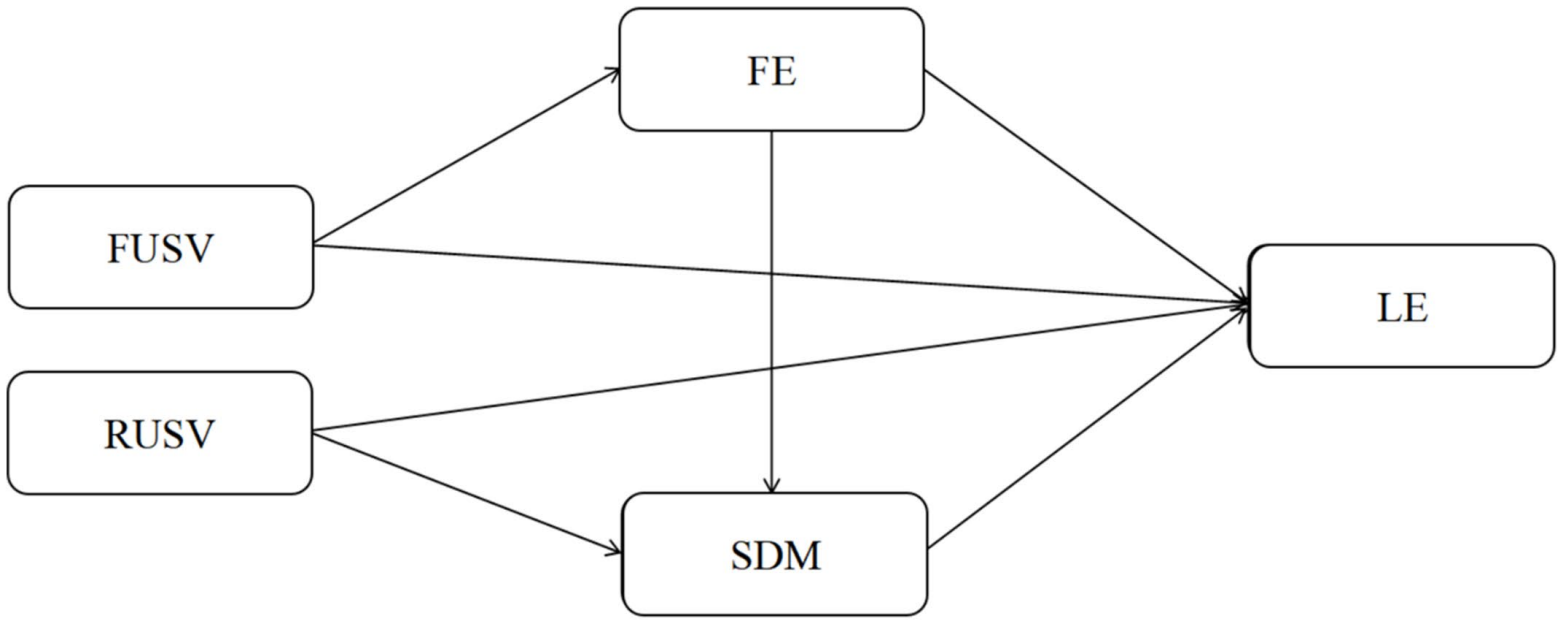
Hypothesis model diagram. Functional use of short video platforms (FUSV); recreational use of short video platforms (RUSV); flow experience (FE); self-determined motivation (SDM); learning engagement (LE).

Concerning the theoretical chain-mediated interaction approach between flow experiences and self-determined motivation, it is possible to formulate a pathway of theorizing. Particularly, the behavior as an user of short video platforms, specifically the type and intensity in the usage, initially contributes to the immediate experiential experience of learners, including flow (high concentration, enjoyment, distortion of time). Further, flow experiences also shape motivation structures among individuals especially how individuals view themselves in terms of autonomy, competence and relatedness. Finally, the experiences as motivators determine long-term learning involvement. This line of argumentation is based on two theoretical premises. To start with, flow is an amalgamation of intense concentration and an optimistic feeling that helps to increase engagement in tasks and their accomplishment, thus developing a sense of ability in people (I can do it) and motivation to do tasks (I want to do it) (Csikszentmihalyi, 1990; Liu et al., 2024). Second, Self-Determination Theory is based on the idea that intrinsic motivation and self-determination are the most appropriate factors that maintain long term engagement on learning activities (Deci and Ryan, 2000). A feeling of autonomy, competence, or relatedness has an experience leads to increased self-determined motivation and hence increases behavioral engagement when the experience is a satisfying one like flow with short video-based learning (Junco, 2012). The theoretical studies have shown that flow experience and self-determination motivation are closely interrelated. The flow experience developed by Csikszentmihalyi (1990) increases the feeling of competence in people, which in turn fosters the intrinsic motivation. The chain-mediated process through which flow experience affects behavioral outcomes by impacting motivational components was verified by recent works by Liu et al. (2024). Yang et al. (2025) also found the chain effect of flow experience as a factor affecting the engagement of online learning by self-determination motivation. According to this theoretical background, the Hypothesis *H4* will be: Flow experience and self-determined motivation have a joint chained influence on the relationship between short video platform use and learning engagement (Figure 1).

## Methods

### Research participants

In this study, the sample of the survey included undergraduate students studying Spanish as a major in various Asian universities. Due to the topic of the research to be conducted on the correlation between the use of the short video platforms and the educational engagement, the sample frame was chosen by purposive as well as convenience sampling methods. Primarily, the research team sent appeals to the departments of colleges of foreign languages and Spanish in China, Japan, South Korea, and Vietnam asking students to take part through online communications. The following criteria were applied: (1) the association of the participant to an organized program at the undergraduate level studying Spanish; (2) the frequent usage of short-video platforms; and (3) the voluntary completion of the questionnaire and valid responses. The number of students that responded to the questionnaire was 412 with 138 males (33.5) and 274 females (66.5) with a mean age of 20.6 years (SD = 1.42). The research team was able to control the grade level and distribution by gender across the universities by this controls ensuring that there were students representing all 4 years of study and this guaranteed that students in first year to the fourth year would be covered. The research was approved by Henan University of science and technology (HAUST-FLS-2025-05-19). Informed consent was written and all the participants gave it before taking part, and the study was performed in line with the ethical considerations of the Declaration of Helsinki. The sample is representative of the short video consumption among undergraduate students studying Spanish in Asia in general and the impact it might have on academic activity in particular.

### Research tools

#### Short video platform usage scale

To quantify the motivations of Spanish undergraduate students in visiting short video platforms, this study took the Uses and Gratifications Theory as the theoretical base referring to the fact that users choose media advert to meet various psychological needs (Katz et al., 1973). Based on the informational acquirement and leisure entertainment dimension that have been suggested in the tourism short video platform research (Qu et al., 2022), two dimensions were formulated in the study: functional use and recreational use. The functional use is defined as the accessing of language learning tools, additional practice scripts, and academic information through short video, and the recreational one defines the level to which students use the short video to relax, entertain, and manage their emotions. The dimensions were assessed using four items (results in a total of 8) measured on a 5-point Likert-scale (1 = strongly disagree, 5 = strongly agree). The scale was good in reliability and validity with Cronbach 0.85 of alpha of functional use and 0.88 of recreational use, which are higher than the assumed psychometric thresholds (Nunnally and Bernstein, 1994). The consistency factor analysis (CFA) showed that the model fits excellently (ch square/df = 2.30, CFI = 0.96, TLI = 0.95, RMSEA = 0.054). Therefore, the scale is useful in measuring unique patterns of usage by Spanish undergraduates using short video sites.

#### Learning engagement scale

In the current study, the Utrecht Work engagement Scale-Student version (UWES-S) designed by Schaufeli et al. (2002) was used to identify the degree of learning engagement in undergraduate students who majored in Spanish and used the Chinese version of the scale that had been altered and applicable in the Chinese context was used to determine learning engagement within the undergraduate students. The UWES-S has undergone a broad range in cross cultural research and it has got 14 items under three dimensions of the learning process; the vigor, dedication and absorption. The responses were noted in terms of a 7-point scale Likert (1 = Never, 7 = Always). Among them, vigor is one that reflects the amount of energy and resilience shown by the students during the learning process, dedication is another character that shows the sense of meaning and enthusiasm that students have had in regard to what they are studying, and absorption is also the total involvement of students in learning processes. The scale was also found to be very reliable and valid with the sample used in this study. The Cronbach alpha of the three subscales that are vigor, dedication and absorption was 0.86, 0.88 and 0.85, respectively, with an overall scale alpha of 0.92, which is greater than the acceptable psychometric standards (Nunnally and Bernstein, 1994). The confirmatory factor analysis outcomes indicated that the three-factor model had a very good fit (*χ*^2^/df = 2.18, CFI = 0.96, TLI = 0.95, RMSEA = 0.051), which indicated that this scale could be used across various cultures with undergraduate Spanish majors in Asia. Moreover, the research that has been done has indicated that student version of UWES is of good reliability and validity among the students of a Chinese university (Zhang and Gan, 2005), which further gives localized justification as to the choice of this instrument in conducting the present study.

#### Flow experience scale

This paper used the Flow State Scale (FSS) and its version (Flow State Scale Short) introduced by Jackson and Marsh (1996) as its theoretical framework. It based on the construct design on the Foreign Language Flow Scale (FLFS) by Wang and Huang (2022) that was used in the context of blended foreign language learning and adapted the items according to the context of using short videos. The updated scale comprised of four central dimensions of Absorption, Time Distortion, Reduced Self-Awareness and Intrinsic Enjoyment and 12 items on a 5-point Likert scale (1 = strongly disagree, 5 strongly agree). The scale in the study sample showed a high level of reliability and construct validity. The 4 dimensions had 0.83–0.89 Cronbach’s alpha with an overall alpha of 0.92, which is above the psychometric acceptable standards (Nunnally and Bernstein, 1994). The four-factor structure had a great model fit based on confirmatory factor analysis (CFA) (*χ*^2^/df = 2.20, CFI = 0.96, TLI = 0.95, RMSEA = 0.050) that demonstrated good structural validity of every dimension. Moreover, Wang and Huang (2022) confirmed the nature of both exploratory and confirmatory factors analysis to prove the presence of feasibility and reliability of the FLFS in the Acts of blended instruction of English. This observation justifies the use of the Flow Experience Scale in the short video learning scenario in Spanish that was conducted in this research.

#### Self-determination motivation scale

In this research, the Academic Motivation Scale (AMS), created by Vallerand et al. (1992), was used as the main tool of determining the degree of self-determined motivation to the Spanish undergraduate students. Items were modified according to local scores of the scale by Guay et al. (2000) and further changes by Chinese researchers. The scale is based on the self-determination theory (Deci and Ryan, 1985) and includes three large categories of motivations, which are intrinsic motivation (knowledge, achievement, and stimulation), extrinsic motivation (identification, introjection and external regulation) and amotivation. The 28-item scale used a 7-point Likert scale (1 = strongly disagree, 7 = strongly agree). There was an excellent internal consistency of the AMS in the study sample. The Cronbach 0.83 to 0.88 intrinsic motivation, 0.79 to 0.85 extrinsic motivation and 0.81 amotivation gave a The Cronbach coefficients value. Section 1 overall scale *α* was equal 0.91, which is above the existing psychometric standards (Nunnally and Bernstein, 1994). It was confirmed by confirmatory factor analysis (CFA) that the good model fit to the seven-factor structure comprised 2.30 (*χ*^2^/df) good fit, 0.95 (CFI) good fit, 0.94 TLI good fit, and 0.054 RMSEA good fit. As it has been established in the previous research, the AMS has high cultural cross application (Cokley, 2000; Huang, 2011) meaning that it is applicable in context of describing the motivational behavior of Spanish language learners.

### Data statistics and analysis

The study employed SPSS 26.0 for descriptive statistics, correlation analysis, and common method bias testing. The moderated mediation model was validated using the SPSS macro Process Model 4.

## Results

### Common method bias examination

In this research one of the things that were used to study and eliminate the possibility of common method bias (CMB). To reduce the potential of social desirability bias, first, anonymity and confidentiality were observed in data collection to reduce the effects of social influences on responses. Second, the one-factor test of CMB was done by Harman (1967). Particularly, all the measured variables were subjected to an exploratory factor analysis (EFA). Findings revealed that the former explained 32.05% of the variance, which is lower than the 50% mark, and thus CMB was not found to have any significant impact on the results. Moreover, the reverse-scored items were also used to the cross-validation of data consistency and reliability. The literature on the topic indicates that common method bias may affect the collected data by the use of a questionnaire, but the various steps undertaken are effective in addressing the effect on the research (Podsakoff et al., 2003). As such, the common method bias was believed to have an insignificant effect on the research results.

### Correlation analysis

**Figure 2:**
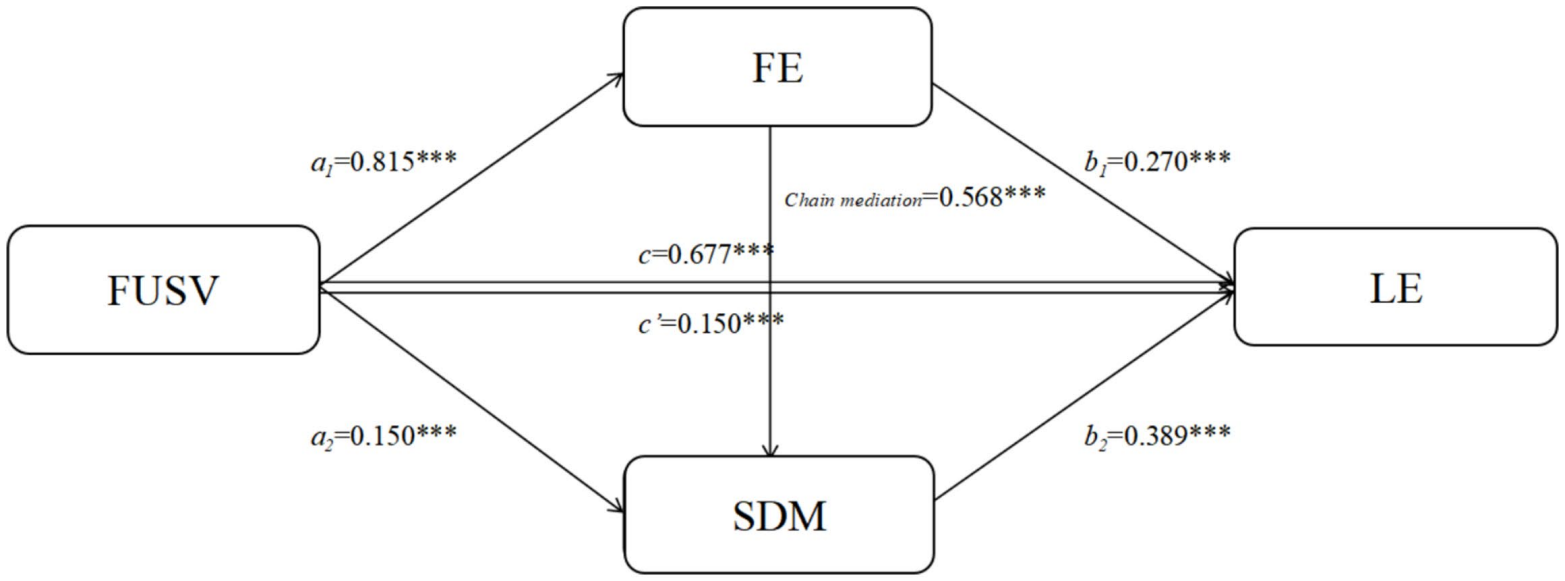
Chain mediation model of FE and SDM in the relationship between FUSV and LE on short video platforms. *p < 0.05; **p < 0.01; **p < 0.001.

Table 1 shows the mean (M), SD, and the correlation between the key variables of the research. The outcome showed that short video platform functional use (*M* = 2.55, SD = 1.07) had significant positive correlation with learning engagement (*M* = 3.36, SD = 1.18), *r* = 0.68, *p* < 0.01. This result implies that Spanish undergraduate students have an increased learning engagement when they use short video websites as their language learning and academic support tools. On the other hand, recreational use of the short video platforms (*M* = 2.43, SD = 0.94) exhibited negative relationship with learning engagement, *r* = −0.30, *p* < 0.01, which indicates that recreational use could be an inhibitor to learning engagement. In addition, flow experience (*M* = 2.37, SD = 0.90) had a positive correlation with learning engagement, *r* = 0.72, *p* < 0.01, with the implication that the flow experience is facilitative to learning engagement. Lastly, self-determined motivation (*M* = 3.28, SD = 1.14) had robust positive correlations with all the other variables, especially the learning engagement (*r* = 0.73, *p* < 0.01) and the flow experience (*r* = 0.83, *p* < 0.01), which further reiterate the primary importance of motivation in promoting the learning process (Figure 2).

**Table 1:** Descriptive statistics and correlation analysis of each variable.

Variable	*M*	SD	1	2	3	4	5
1. FUSV	2.553	1.065	1				
2. RUSV	2.430	0.941	−0.511**	1			
3. LE	3.364	1.176	0.677**	−0.301**	1		
4. FE	2.368	0.898	0.816**	−0.497**	0.716**	1	
5. SDM	3.278	1.139	0.789**	−0.395**	0.732**	0.833**	1

*p < 0.05; **p < 0.01; **p < 0.001.

**Figure 3:**
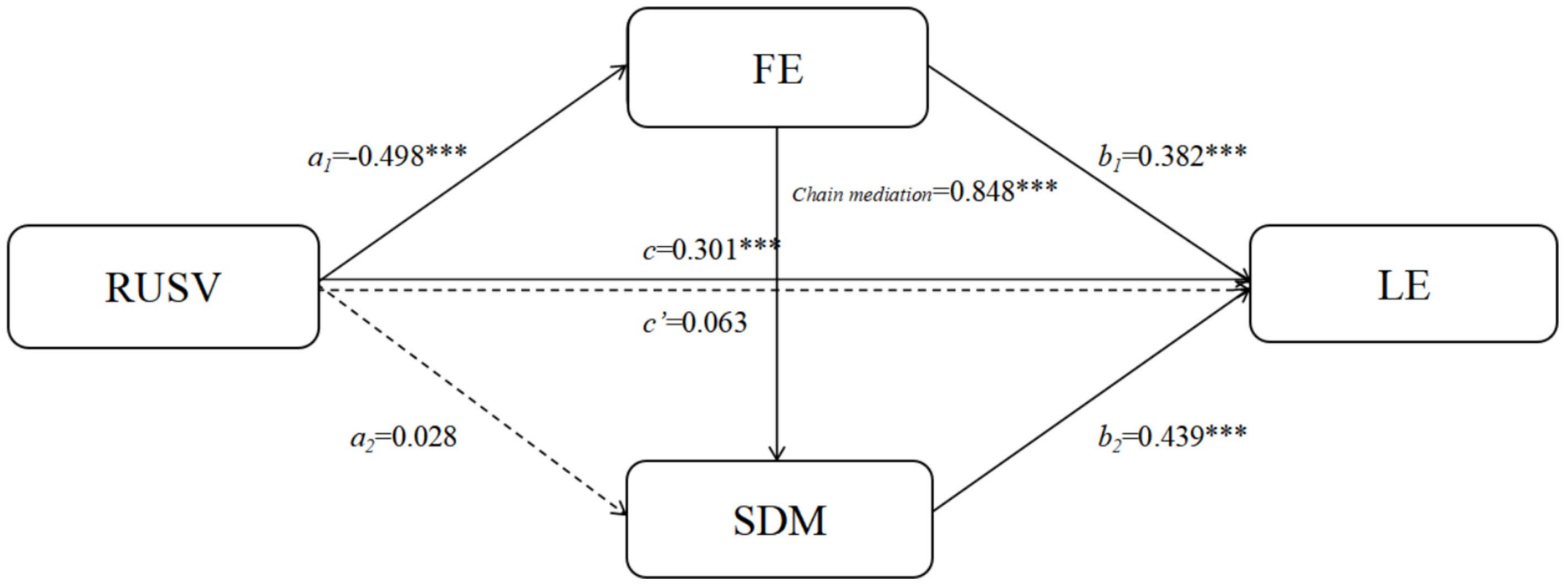
Chain mediation model of FE and SDM between RUSV and LE on short video platforms. *p < 0.05; **p < 0.01; **p < 0.001.

The findings of the chained mediation analysis which assesses the significance of flow experience and self-determined motivation in the association between the functional use of short video platforms and learning engagement is published in Table 2. The results showed that short video platforms predict learning engagement significantly and positively, 45.9% of the variance was explained with 2 = 0.68, *p* = 0.001 as the coefficient of determination. Also, functional use was a significant predictor of flow experience (=0.82, *p* < 0.001) and self-determined motivation (=0.33, *p* < 0.001), which indicated that functional use affected learning engagement through strengthening the two mediating variables. Later analysis found out flow experience played a significant role in the mediation of the relationship between functional use and learning engagement (4.22 = 0.27, *p* = 0.001), whereas self-determined motivation partially mediated this relationship (4.22 = 0.39, *p* = 0.001). The bootstrap outcome of the mediating effects in each of the paths is shown in Table 3. Functional use had a significant total effect on the learning engagement (=0.68) and the whole effect was also confirmed to be true by the indirect pathways. Particularly, the functional and indirect effects on the learning engagement included flow experience (2 = 0.22, *p* < 0.001) and self-determined motivation (2 = 0.13, *p* < 0.001). Moreover, the mediating impact of flow experience on self-determined motivation was also sequential (chain) and significant (*β* = 0.18, *p* = 0.001), which only increased the effect of functional use on learning engagement. Collectively, these results indicate flow experience and self-determined motivation are important sequential mediating factors in the relationship between the functional use and learning engagement, which provides a possible mechanism that platform use can impact learning outcomes (Figure 3).

**Table 2:** Testing the chain mediation effect of FE and SDM on FUSV and LE in short video platforms.

Dependent variable	Independent variable	*R*	*R* ^2^	*F*	*β*	*t*
LE	FUSV	0.677	0.459	115.186***	0.677	18.583***
FE	FUSV	0.816	0.667	271.739***	0.815	28.509***
SDM	FUSV	0.855	0.731	276.208***	0.326	7.332***
	FE				0.568	12.760***
LE	FUSV	0.761	0.580	112.022***	0.150	2.529***
	FE				0.270	4.093***
	SDM				0.389	6.278***

*p < 0.05; **p < 0.01; **p < 0.001.

**Table 3:** Bootstrap test of the chain mediation effect of FE and SDM on FUSV and LE in short video platforms.

Path	Effect	BootSE	BootLLCI	BootULCI
Total effect	0.677	0.036	0.606	0.749
Direct effect	0.150	0.059	0.033	0.266
Indirect effects	0.527	0.047	0.435	0.617
FUSV→FE → LE	0.220	0.051	0.119	0.319
FUSV→SDM → LE	0.127	0.027	0.078	0.182
FUSV→FE → SDM → LE	0.180	0.032	0.120	0.245

The outcomes of the chained mediation analysis that focused on the functions of flow experience and self-determined motivation in the association of recreational utilization of short video platforms and learning engagement are shown in Tables 4, 5. In terms of direct effects, recreational consumption of short video sites was a strong and significant predictor of learning engagement, 907 = −0.30, *p* = 0.001 and it explained almost 9.1 percent of its variance. Flow experience was also strongly and adversely predicted by recreational use *β* = −0.50, *p* = 0.001. Flow experience in turn had significant and positive prediction of self-determined motivation, *β* = 0.85, *p* < 0.001, which in turn was a predictor of learning engagement, *β* = 0.38, *p* < 0.001, and 44, *p* < 0.001. But the effect of recreational use on self-determined motivation was not significant, *β* = 0.03, *t* = 0.87 *p* > 0.05, so the enhancements of self-determined motivation was mainly based on the mediating effect of flow experience.

**Table 4:** Testing the chain mediation effect of FE and SDM between RUSV and LE on short video platforms.

Dependent variable	Independent variable	*R*	*R* ^2^	*F*	*β*	*t*
LE	RUSV	0.302	0.091	13.605***	−0.301	−6.378***
FE	RUSV	0.500	0.250	45.434***	−0.498	−11.628***
SDM	RUSV	0.834	0.696	232.731***	0.028	0.870
	FE				0.848	26.865***
LE	RUSV	0.759	0.576	110.355***	0.063	1.686
	FE				0.382	6.152***
	SDM				0.439	7.486***

*p < 0.05; **p < 0.01; **p < 0.001.

**Table 5:** Bootstrap test of the chain mediating effect of FE and SDM on the relationship between RUSV and LE on short video platforms.

Path	Effect	BootSE	BootLLCI	BootULCI
Total effect	−0.301	0.047	−0.394	−0.208
Direct effect	0.063	0.037	−0.011	0.136
Indirect effects	−0.364	0.038	−0.441	−0.291
RUSV→FE → LE	−0.191	0.035	−0.263	−0.124
RUSV→SDM → LE	0.012	0.013	−0.013	0.038
RUSV→FE → SDM → LE	−0.186	0.030	−0.248	−0.129

Table 5 displays the bootstrap findings of the tests of the indirect effects of every mediation pathway. The overall impact of recreational use on learning involvement was negative and significant, effect = −0.30, 95% CI [−0.39, −0.21], but not the direct impact which was invigilating, effect = 0.06, 95% CI [0.01, 0.14]. This trend indicates that recreational use is the major factor that affects learning involvement in an indirect manner. Namely, the indirect effect through flow experience alone was significant, effect = −0.19, CI [−26, −12], but the indirect effect, through self-determined motivation alone, was non-significant, effect = 0.01, CI [−1, −0.04]. The most important effect that was found was the negative chained indirect effect that recreational use had via the sequential path flow experience [self-determined motivation], effect = −0.19, 95%CI [−0.25, −0.13].

To conclude, undergraduate students studying in Spain are adversely impacted by the recreational use of short video platforms in learning. This effect mainly works with a mechanism of this diminished flow experiences lowering self-determined motivation which subsequently indirectly lowers learning engagement.

## Discussion

The present research paper investigated how the use of short video platforms can affect the learning process of Spanish undergraduate enrollment students in Asia, in terms of their mechanisms underlying the flow experience and self-determined motivation concepts. The results indicate that the functional use of short video platforms has a significant positive effect on the engagement of the learning process, in the first place, it improves the flow experiences of learners and learners having self-determined motivation. Recreational use, on the contrary, has a negative impact on learning engagement, which is mainly dominated by a reduction in flow experiences and undermining of self-determined motivation. These results are discussed more in detail in the following sections with a reference to the context of the previous research and illuminated limitations and implications of the study to future research.

To begin with, the functional use of short video platforms and learning engagement in this study were found to have a significant positive correlation. The applied use was demonstrated to have an indirect boost on learning engagement through activating flow experiences and bolstering self-determined motivation. Particularly, the immersion and concentration, which lies at the heart of flow experience, were made easier by the structured, educational and goal-oriented character of content used by Spanish undergraduates as the means of learning using the short video platforms (Csikszentmihalyi, 1990). Additionally, quick feedback offered by these sites created a feeling of competence and self-efficacy hence aiding greater degrees of self-determined motivation (Deci and Ryan, 2000). These findings can be considered in congruity with previous research; e.g., Ye et al. (2024) showed that short videos, used in the sphere of education, could be effectively used to boost the level of student engagement and academic performance by boosting the level of learning motivation and the active participation rates. Combined, these results imply that the practical application of short video platforms is a potential addition to conventional classroom training, and can have the effect of enhancing learning activity among the student population on the undergraduate level in Spanish.

Nevertheless, another important negative effect that the study found in the recreational use of short video platforms is the reduction of both flow experiences and self-determined motivation as a result of the use of these kinds of platforms. Students are more likely to watch any video longer and disjointed when they use platforms of short videos more as recreational sources, than as learning ones, which usually leads to distraction and unproductive time management. In comparison to functional use, recreational use does not have specific learning goals and carefully organized material since videos tend to be created with the purpose of entertainment and amusement instead of in-depth learning that is of the essence of long-term learning. According to the flow theory, optimum flow experiences are achieved when learners feel that the task presented and their abilities are well matched (Csikszentmihalyi, 1990). Such conditions would not be found in recreational use, which results in the fact that cognitive resources are diverted and the level and quality of learning decline (Liu et al., 2024). These results are similar to those reported by Wong et al. (2024), who have argued that recreational use is likely to consumed excessive study time by students hence frustrating learning engagement and educational attainment. Combined, the findings indicate that, despite the tremendous attractiveness of short video sites toward recreational activities, they can also be detrimental to the academic growth of students in that the viability of the deep and prolonged engagement is weakened.

This study revealed that both flow experience and self-determined motivation were important in examining their chain-mediated role to the relationship between functional short video use and the engagement in self-directed learning. First, flow experience boosts interest in that it brings about concentration, pleasure, and distortion of time thus enabling learners to be fully engaged in learning activities (Lin et al., 2025; Oliveira et al., 2022). Second, increased self-determined motivation is another way of reinforcing engagement. One of the primary explanations for maintaining engagement in learning, according to self-determination theory, is intrinsic motivation and a sense of accomplishment in the fundamental needs of individuals; competence, relatedness, and autonomy (Ojo et al., 2024; Yang et al., 2025). When the needs are met by short video platforms, then they trigger autonomous learning motivation and increase student involvement. In particular, a platform that emphasizes the competence and autonomy of learners and presents the content about the language through it supports the level of intrinsic motivation and, consequently, leads to a more productive involvement in academic activities. These results, collectively, imply that the functional utilization of short video services facilitates more learning by using the two mechanisms as middlegrounds flow experience and self-determined motivation (Wu, 2024).

Nonetheless, recreational use has a negative impact in various ways. Frivolous and lightweight content does not involve intensive inquiry often helps avoid the deep concentration of attention and personal pleasure of flow experiences (Lin et al., 2025). Though this use might offer certain temporary gratification, it is mainly more to satisfy the external motivation needs (e.g., leisure and relaxation) instead of intrinsic learning needs. In the long run, this incompatibility may cause the conflict between entertainment and academic activities, which would reduce the motivation to learn among students (Ojo et al., 2024; Yang et al., 2025). The current results showed that recreational use reduces self-determined motivation among the students indirectly through inhibited flow experiences, which further decreases the learning interest. This finding is in line with previous studies that indicated that compulsive recreational short videos viewing may have a detrimental impact on academic performance (Junco, 2012).

### Limitations and future directions

Although this study sheds light on the psychological mechanisms underlying short video platform usage, several limitations should be acknowledged. First, the sample was limited to Spanish-speaking undergraduates in Asia. Future research could broaden the scope by including students from other language backgrounds, academic disciplines, and cultural contexts to enhance the generalizability of the findings. Second, data collection relied exclusively on self-reported questionnaires, which are subject to biases such as social desirability and inaccurate recall, potentially limiting the precision of the results. To address this, future studies could employ experimental designs, longitudinal approaches, or behavioral data analysis to provide more objective evidence of the impact of short video platform usage on learning engagement. Third, this study did not consider external influences such as instructors’ pedagogical design or the quality of course content, both of which may significantly affect learning engagement. Future research should integrate these external variables into the analytical framework to examine how they interact with short video usage. By addressing these limitations, subsequent studies can provide a more comprehensive understanding of the relationship between short video platforms and student learning engagement.

## Conclusion

This paper develops a theoretical model that combines the Uses and Gratifications Theory, Flow Theory, and Self-Determination Theory and explains the impact of the use of short-form videos platforms on learning engagement. The explanatory power of this integrated framework is confirmed by the results. The Uses and Gratifications Theory is used to understand why students make various use types. Flow Theory explains that functional use helps to provide immersive experience through the development of challenge-skill balance, clear goals, and feedback. Self-Determination Theory demonstrates that these experiences, which help individuals fulfill a need of autonomy, competence and relatedness, boost self-determined motivation, hence increasing engagement. The combination of these theories creates an entire chain of explanation: use choice → experience generation→ motivation formation→ outcome manifestation.

## Data Availability

The raw data supporting the conclusions of this article will be made available by the authors, without undue reservation.
